# Lithiase géante sur enterocystoplastie

**DOI:** 10.11604/pamj.2014.19.357.5510

**Published:** 2014-12-08

**Authors:** Khalid Elmortaji, Ghassane Elomri, Saad Bennani, Redouane Rabii, Rachid Aboutaib, Fethi Meziane

**Affiliations:** 1 Service d'Urologie CHU ibn Rochd de Casablanca, Casablanca, Maroc

**Keywords:** Enterocystoplastie, lithiase, tumeur, enterocystoplasty, lithiasis, tumor

## Abstract

La formation des lithiases est une complication fréquente des entérocystoplasties après cystectomie radicale pour tumeur de vessie infiltrante. Le délai d'apparition dépend des facteurs de risque favorisants notamment les infections urinaires. Néanmoins la survenue de lithiase géante sur néovessie reste exceptionnelle, seulement 5 cas ont été rapportés dans la littérature. Nous rapportons dans ce travail, le cas d'une lithiase géante compliquant une entérocystoplastie chez un malade suivi pour tumeur de vessie infiltrante.

## Introduction

La formation des lithiases est une complication fréquente des entérocystoplasties après cystectomie radicale pour tumeur de vessie infiltrante. Son incidence varie entre 12 et 52,4% [1]. Le délai d'apparition dépend des facteurs de risque favorisants notamment les infections urinaires. Néanmoins la survenue de lithiase géante sur néovessie reste exceptionnelle, seulement 5 cas ont été rapportés dans la littérature [2].

## Patient et observation

Mr BM âgé de 65 ans, agriculteur, ancien tabagique chronique sevré depuis 1999, est suivi pour tumeur de vessie infiltrante, ayant bénéficié d'une cysto-prostatectomie en 2000. Le suivi post opératoire était sans particularité pendant les 4 premières années, puis le patient a été perdu de vue. Il est réadmis aux urgences urologiques en Décembre 2012 pour douleur lombaire bilatérale, constipation et sensation de pesanteur pelvienne. A l'examen clinique: patient est en bon état général, les fosses lombaires légèrement sensibles et on note la présence d'une voussure hypogastrique dure à la palpation, immobile. Cette masse arrive au sacrum et comprime le rectum au toucher rectal.

**Figure 1:**
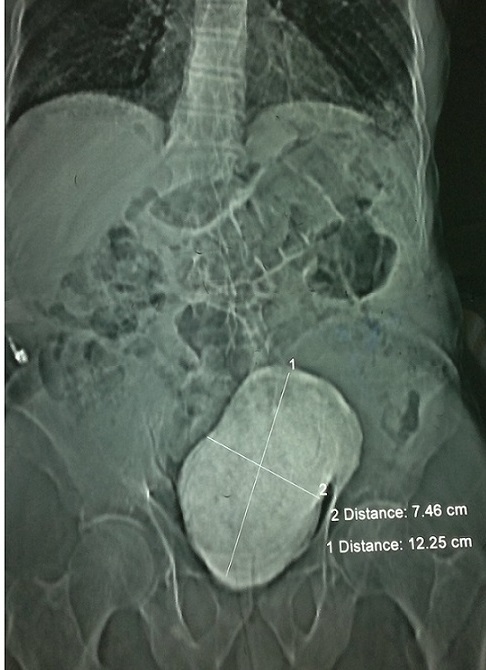
AUSP montrant une opacité de tonalité calcique occupant tout le pelvis de 12,25x7, 46 cm

**A la radiographie sans préparation:** on note opacité de tonalité calcique ovalaire prenant tout le pelvis mesurant 12,25 x 7,46 cm. (Figure 1).

**A l’échographie rénale et pelvienne:** les deux reins sont de taille normale bien différenciés, avec une urétéro-hydronéphrose bilatérale minime, et présence d'une image hyperéchogène avec un cône d'ombre postérieur occupant tout le pelvis.

**La fonction rénale** est normale.

**Figure 2:**
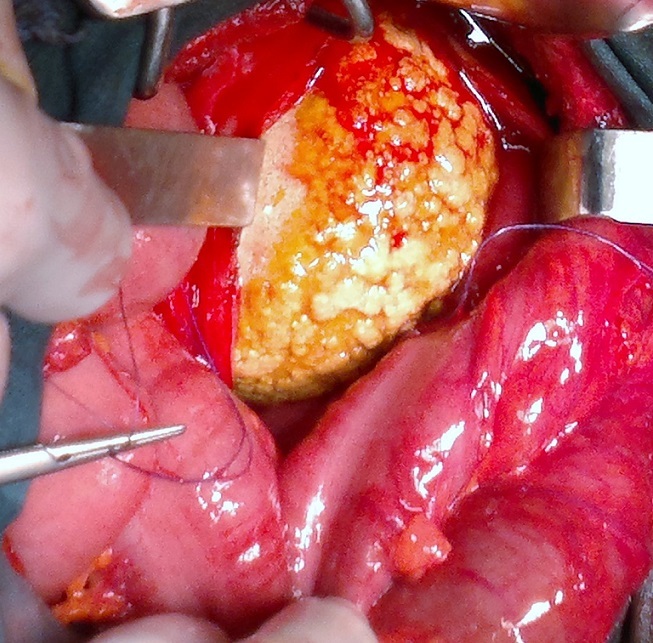
Lithiase géante sur entérocystoplastie en per opératoire

**Figure 3:**
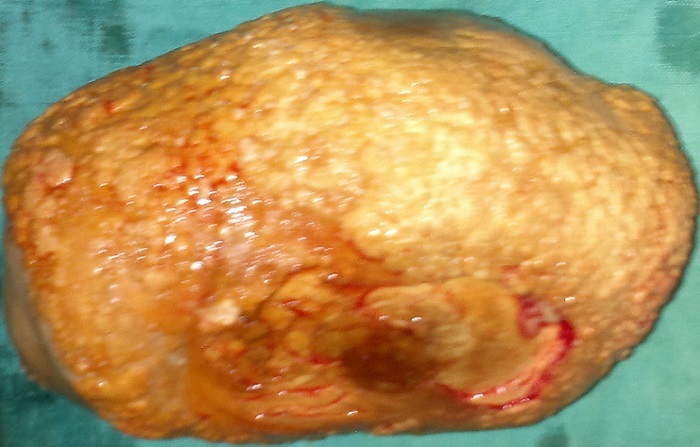
Image de la lithiase géante après néocystolithotomie

**L'ECBU** montre une infection urinaire à Echerichia.coli sensible à l'imipenème. Le traitement a consisté, après une antibiothérapie adaptée, en une entérocystolithotomie avec extraction d'une lithiase géante pesant 1200 gramme de consistance solide (Figure 2, Figure 3). A l’étude spectrophotométrique: la lithiase est à base d'oxalate de calcium et de magnésium. Les suites opératoires sont simples.

## Discussion

La cystectomie radicale avec entérocystoplastie ou Bricker représente le gold standard dans le traitement des tumeurs de vessie infiltrant le muscle depuis 1950 [3]. Les complications à long terme sont dominées par les infections urinaires à répétitions avec un taux de 25% [4], et les lithiases sur entérocystoplastie avec un taux qui varie entre 12 et 52,4% [1]. Néanmoins, la survenue de lithiase géante reste une complication exceptionnelle; seulement 5 cas ont été rapportés dans la littérature, dont la plus volumineuse pesait 940 grammes [2]. Parmi les facteurs de risque incriminés dans la formation de lithiases sur enterocystoplastie, on trouve les infections urinaires à répétition, Néanmoins 75% des patients qui présentent des infections urinaires n'ont pas développé de lithiase ce qui suggère que l'infection urinaire n'est pas le principal facteur favorisant la constitution des lithiases sur enterocysoplastie plutôt qu'une conséquence de celle-ci. [5]. On cite également, le rôle primordial du taux urinaire en oxalate du calcium, magnésium, et du phosphate dans la lithogénèse; Selon Takeda et al [6] le nombre de facteurs de risque urinaire influence l'incidence des lithiases sur néovessie, 80% des patients qui ont deux facteurs de risque ou plus ont développé une lithiase sur néovessie. D'autres facteurs peuvent être incriminés dans la formation des lithiases sur enterocystoplastie notamment [6]: les modalités de drainage d'urine; La présence de corps étranger à l'intérieur de la néovessie; L'immobilité joue un rôle dans la formation de lithiases par la stase des urines; La constitution du mucus en calcium et en magnésium.

Plusieurs études ont montré que l'agrandissement vésical ou le remplacement vésical n'a pas augmenté l'incidence de lithiase [7]. Néanmoins, la fréquence des infections urinaires et des lithiases post-opératoires sont à l'origine de l'abandon progressif des entérocystoplasties au profit des dérivations externes. Quant au diagnostic positif: l'examen clinique est souvent pauvre et non spécifique. L'arbre urinaire sans préparation (AUSP) et l’échographie abdominale posent le diagnostique dans 95% des cas. L'urographie intra-veineuse ou l'uroscanner permettent une exploration panoramique de tout l'appareil urinaire et une analyse spécifique de la lithiase: localisation, nombre, taille, densité et le retentissement sur le haut appareil urinaire. Le traitement des lithiases sur enterocystoplastie dépend de la taille des lithiases et de la nature de la dérivation urinaire utilisée, cependant la néocystolithotomie reste le traitement de choix dans les lithiases volumineuses [8], comme c’était le cas pour notre patient.

D'autres modalités thérapeutiques peuvent être envisagées: Lithotritie endoscopique au laser par l'introduction d'un fibroscope souple à travers le système de dérivation continente et fragmentation de la lithiase au laser Holmium pour les petites lithiases. [9]; Plus récemment, l'abord percutané, avec ou sans lithotripsie adjuvante, a été proposé comme traitement de première ligne en cas de néo-vessie. L'efficacité immédiate et en terme de récidive est identique pour un temps opératoire plus court et des suites opératoires plus simples [10]; Pour la lithotritie extra corporelle, son efficacité reste limitée dans les lithiases sur enterocystoplastie [10]; Enfin, la prévention des lithiases sur entéroystoplastie reste difficile, elle repose essentiellement sur le traitement des infections urinaires; une hydratation adéquate (2L/jours) et un régime alimentaire riche en fibre alimentaire [6].

## Conclusion

La survenue de lithiase géante sur entérocystoplastie reste une complication exceptionnelle, dont l'infection urinaire à répétition et le mode de drainage sont les principaux facteurs incriminés; son traitement dépend avant tout de la taille de lithiase et l'expérience du chirurgien.
